# Polyethylene glycol 20 kDa-induced vacuolation does not impair phagocytic function of human monocyte-derived macrophages

**DOI:** 10.3389/fimmu.2022.894411

**Published:** 2022-07-28

**Authors:** Anne Schoenbrunn, Kerstin Juelke, Birgit M. Reipert, Frank Horling, Peter L. Turecek

**Affiliations:** ^1^ BIH Center for Regenerative Therapies and Institute of Medical Immunology, Charité Universitätsmedizin, Berlin, Germany; ^2^ R&D Baxalta Innovations GmbH, part of Takeda, Vienna, Austria

**Keywords:** monocyte-derived macrophages, PEGylated recombinant coagulation factor VIII, polyethylene glycol, rurioctocog alfa pegol, vacuolation, vacuolization

## Abstract

Conjugation to polyethylene glycol (PEG) is commonly used to enhance drug delivery and efficacy by extending the half-life of the drug molecule. This has important implications for reducing treatment burden in diseases that require chronic prophylaxis, such as hemophilia. Clearance of PEG molecules with high molecular weights (≥ 40 kDa) has been reported to cause cellular vacuolation in mammals. Rurioctocog alfa pegol (PEGylated recombinant coagulation factor VIII) contains a 20-kDa PEG. This study investigated the effects of exposure to 20-kDa PEG (10 μg/ml to 10 mg/ml) on the morphology and function of human monocyte-derived macrophages (MDMs) *in vitro*. Exposure to PEG for 24 hours was associated with significant vacuolation only at concentrations of 1 mg/ml or more, which far exceed the levels associated with clinically relevant doses of rurioctocog alfa pegol. Immunofluorescence staining of PEG was detected in the cytoplasm of MDMs, indicating uptake into the cells. No impairment of MDM phagocytic activity (ability to ingest fluorescently labeled *Escherichia coli*) was observed with 24-hour exposure to PEG, even at concentrations associated with significant vacuolation. Furthermore, PEG exposure did not have significant effects on cytokine secretion in resting or lipopolysaccharide-stimulated MDMs, or on the expression of cell surface markers in stimulated MDMs. Cell viability was not affected by 24-hour exposure to PEG. In conclusion, vacuolation of human MDMs after exposure to 20-kDa PEG only occurred with PEG concentrations far in excess of those equivalent to clinically relevant doses of rurioctocog alfa pegol and did not affect MDM viability or functionality. Together, these results support the concept that PEG-mediated vacuolation is an adaptive cellular response rather than a toxic effect.

## Introduction

Polyethylene glycol (PEG) is an inert, nondegradable, water soluble, amphiphilic polyether comprising repeating units of ethylene glycol (1). It is widely used in everyday consumer products, as well as an excipient in pharmaceuticals and an emulsifier in food preparations (1, 2). PEGylated therapeutics have been in use since the 1990s (1, 2). These therapies contain one or more PEG polymers covalently attached to a drug molecule to enhance drug delivery and efficacy (2, 3). Rurioctocog alfa pegol (PEGylated recombinant coagulation factor VIII) is a full-length recombinant factor VIII (rFVIII) conjugated to 20-kDa branched PEG molecules, each consisting of two 10-kDa chains (2). The PEGylation of rFVIII prolongs its half-life in circulation, allowing for reduced treatment burden and maintenance of higher trough levels in people with hemophilia (2, 4, 5).

The main elimination route for PEG molecules is *via* the kidneys, triggered by its water solubility, with some clearance also *via* the liver (2). A small proportion of PEG can also be taken up by cells of the mononuclear phagocyte system (2). These phagocytic cells routinely engulf particles and form intracellular vacuoles as part of their normal process for the removal of foreign materials (6). PEG and PEG-containing therapies therefore have the potential to lead to vacuolation of macrophages after repeated treatment with high doses (2). In mammals, clearance of PEG molecules by macrophages has been reported to cause vacuolation of cells in different tissues as part of the physiological elimination process at high cumulative doses (2, 7). Vacuolation has not been associated with tissue or organ damage in animal studies and there is no evidence of PEG-related safety issues in clinical studies (1, 2, 6, 8, 9).

PEG polymers with median molecular weights of 40 kDa or higher exhibit slower renal elimination than smaller PEGs and therefore have greater potential for uptake by phagocytic cells (2, 9). Correspondingly, PEG-mediated vacuolation has generally been observed only with PEG molecules that have molecular weights of at least 30 kDa (6). The final degradation product of rurioctocog alfa pegol after catabolism of the rFVIII protein is a 20-kDa PEG-acid (PEG2ru20KCOOH) (2). The concentration of 20-kDa PEG required to induce macrophage vacuolation, and the consequences of this process, are not well understood. This study aimed to evaluate the effects of exposure to 20-kDa PEG *in vitro* on the morphology and function of human monocyte-derived macrophages (MDMs). Typical prophylactic doses of PEGylated rFVIII in patients with severe hemophilia A would result in PEG plasma levels of around 0.1 μg PEG/ml blood (10). In line with the aim of toxicological studies to demonstrate an effect of the investigated compound a concentration 10 μg/ml was chosen as the lowest PEG concentration, which is still 100-fold above the expected plasma concentration after a single prophylactic dose of rFVIII. Based on reports of vacuolation of macrophages after exposure to high concentrations of polymers, a concentration of 10 mg/ml PEG was selected as positive control to induce vacuolation in MDMs. Choice of this cell type and design of the study was based on the results, particularly the elimination patterns, of a previously published distribution study in rats with a radiolabeled PEG-rFVIII where tritium (T; ^3^H) labelled PEG was used for conjugation with full length rFVIII (2).

## Materials and methods

### Derivation of human monocyte-derived macrophages (MDMs)

Peripheral blood mononuclear cells (PBMCs) were isolated from buffy coats from 27 healthy donors (German Red Cross [DRK], Berlin, Germany) using density gradient centrifugation (11, 12). CD14+ monocytes were then isolated from the PBMCs using MACS positive selection technology (Miltenyi Biotec). After isolation, CD14+ monocytes were cultured for 7 days at 37°C and 5% CO_2_ in the presence of 50 ng/ml recombinant human macrophage colony-stimulating factor (M-CSF; PeproTech) to generate resting MDMs. The MDMs were plated into 24-well, flat-bottomed plates and rested for 24 hours before use in the PEG-exposure experiments detailed below.

### Exposure of MDMs to PEG

PEG2ruCOOH-20K (20-kDa PEG; Nektar Therapeutics) resolved in a volume of up to 100 μl sterile phosphate buffered saline (PBS) at room temperature was added to the 24-well plate containing resting, adherent MDMs in 1 ml medium (RPMI-1640 [Biochrome] + L-glutamine, 100 U/ml penicillin and 0.1 mg/ml streptomycin + 10% human AB serum [PAN-Biotech]) to reach a final PEG concentration of 10 μg/ml, 100 μg/ml, 1 mg/ml or 10 mg/ml. As a control, 100 μl of sterile PBS was added instead of the PEG solution.

### Assessment and semiquantitative analysis of PEG-mediated vacuolation

To evaluate vacuolation of MDMs after exposure to PEG, MDMs were incubated with PEG at concentrations of 10 μg/ml, 100 μg/ml, 1 mg/ml and 10 mg/ml or with PBS control for 24 hours. Exposure of MDMs to 20-kDa PEG for 24 hours was chosen because organ distribution studies with tritiated (^3^H; T) rFVIII-PEG, where the radiolabel was in the PEG moiety, showed maximum levels of PEG at 24 hours after application in organs rich in phagocytic cells such as liver, intestine and spleen (unpublished data). MDMs were washed twice with PBS prior to fixation in 2.5% glutaraldehyde in 0.1 M sodium cacodylate buffer solution at 4°C overnight and post-fixation in 1% osmium tetroxide/0.8% K_4_[Fe(CN)_6_] in 0.1 M sodium cacodylate buffer solution for 1.5 hours. The fixed cells were then serially dehydrated in ethanol and embedded in epoxy resin. Ultra-thin sections (70 nm) were cut using an Ultracut S microtome (Leica), collected on copper grids and stained with uranyl acetate and lead citrate for analysis by transmission electron microscopy (TEM; Leo EM 906 [Zeiss]). Semi-thin sections (0.5–1 μm) were cut using a Mikrotom RM 2065 (Leica) and stained with methylene blue. These sections were analyzed by light microscopy using an Axio Scope (Zeiss) at a magnification of 63×. Four to ten images were taken, covering the complete area of the section. Each image belonging to one semi-thin section was analyzed with ImageJ software (13). All MDMs visible on the picture were counted and marked with a white spot. All vacuolated MDMs were counted and scored according to vacuolation stage (stage A: 1–3 small vacuoles; stage B: > 3 small vacuoles or ≥ 1 big vacuole [approximately 5% of the cell’s internal diameter]; stage C: vacuolation of ≥ 75% of cell volume). All data were added to a prepared calculation table in Excel and the mean values (% of vacuolated cells; and % of stage A, B and C among vacuolated cells) from all images of one semi-thin section were calculated and plotted. Graphical representations were generated with Graphpad Prism5 Software. An example of semiquantitative analysis of cell vacuolation is shown in **Supplementary Figure 1**.

To evaluate the granularity of MDMs after exposure to PEG, MDMs were incubated with PEG at a concentration of 10 mg/ml or PBS control for 24 hours. The MDMs were then resuspended in 200 μl sterile PBS and analyzed for side scatter channel (SSC) using an LSR II flow cytometer (BD Biosciences). DAPI (4’,6-diamidino-2-phenylindole; 1:250) was added directly before flow cytometric analysis to exclude dead cells.

### Assessment of PEG uptake

Confocal microscopy was used for detection of intracellular PEG. For immunofluorescence (IF), MDMs were incubated on glass coverslips with PEG (10 mg/ml) or PBS control for 24 hours. They were then washed twice with PBS and fixed in 3% paraformaldehyde for 20 min. All steps were performed at room temperature unless otherwise indicated. After three washes in PBS, the cells were permeabilized in 1% Triton X-100 for 15 min and washed again. The MDMs were incubated with 5% goat serum before incubation with primary antibodies (rat anti-PEG [ab94764] and mouse anti-human CD68 [ab955]; Abcam) at 4°C overnight. The MDMs were then incubated with secondary antibodies (goat anti-rat IgG H+L Alexa Fluor 488 [A-11006] and goat anti-mouse IgG H+L Alexa Fluor 594 [A11005]; Thermo Fisher) at 4°C overnight, washed in PBS and incubated with DAPI for 10 min to label cell nuclei. The coverslips were mounted onto object plates and confocal microscopy was performed using an LSM 780 confocal microscope (Zeiss).

For live-cell imaging, MDMs were transferred into a μ-Slide 8-well ibiTreat chamber slide (Ibidi) 24 hours prior to exposure to PEG. They were then incubated for 4 hours with PEG coupled to Alexa Fluor 594 (PEG-A594; Takeda) at a concentration of 1 mg/ml or with PBS control. The chamber slide was then moved to the incubation chamber (37°C and 5% CO_2_) of an LSM 780 confocal microscope (Zeiss) for 26 hours.

### Functional assays

Phagocytic activity of MDMs incubated with PEG 10 μg/ml or 10 mg/ml or PBS control for 24 hours was assessed using the PHAGOTEST™ kit (BD Biosciences), following the manufacturer’s instructions with modification for adherent cells. Samples were analyzed using Calibur or LSR II flow cytometers (BD Biosciences). The test enables distinguishing FITC labeled *Escherichia coli* (*E.coli*) bound to the surface of the MDMs from those phagocytosed.

Cytokine secretion was measured using a multiplex assay kit (V-PLEX Custom Human Cytokine Proinflammatory Panel 1 [6-Plex]; Meso Scale Discovery). MDMs were incubated with PEG at concentrations of 10 μg/ml, 100 μg/ml, 1 mg/ml and 10 mg/ml or PBS control for 24 hours. They were then washed twice with PBS and incubated for another 24 hours, during which half of the wells were exposed to 50 ng/ml lipopolysaccharide (LPS; Enzo Life Sciences). Supernatants were collected and stored at −80°C before analysis of interleukin (IL)-1β, IL-6, IL-8, IL-10, IL-12p70 and tumor necrosis factor alpha (TNFα) concentrations according to the manufacturer’s instructions using a MESO QuickPlex SQ 120 (Meso Scale Discovery).

To examine the effect of PEG on expression of cell surface activation markers, MDMs were incubated with PEG at concentrations of 10 μg/ml, 100 μg/ml, 1 mg/ml and 10 mg/ml or PBS control for 24 hours. They were then washed twice with PBS and incubated for another 24 hours, during which half of the wells were exposed to 50 ng/ml LPS. Following resuspension in PBS, the MDMs were incubated with primary antibodies (anti-human CD80 PE [557227] and anti-human CD86 APC [555660]; BD Biosciences; anti-human HLA-DR PacBlue [B36291]; Beckman Coulter) at 4°C for 10 min. The MDMs were then washed, resuspended in PBS and analyzed using an LSR II flow cytometer (BD Biosciences).

The Annexin V Apoptosis Detection Kit APC (eBioscience) was used to evaluate viability of MDMs after exposure to PEG at concentrations of 10 μg/ml, 100 μg/ml, 1 mg/ml and 10 mg/ml or PBS control for 24 hours, according to the manufacturer’s instructions. MDMs that were negative for Annexin V and propidium iodide staining were defined as vital cells.

### Data analysis

Flow cytometry raw data files were analyzed using FlowJo 9.8.2 software. Multiplex assay raw data were analyzed with a four-parameter logistic curve of each standard using the Discovery Workbench 4.0 software. Data processing was performed using Microsoft Excel and GraphPad Prism 5 software.

For functional assays, results for PEG-treated MDMs were calculated as a percentage of the corresponding control groups. An effect was considered treatment-specific and relevant if the value of the effect was equal to or higher than 200% (increase) or lower than 50% (decrease). These limits were set based on the acceptance values for the experimental variability (coefficient of variation ≤ 20%).

## Results

### Effect of PEG exposure on vacuolation of MDMs

**Figure 1:**
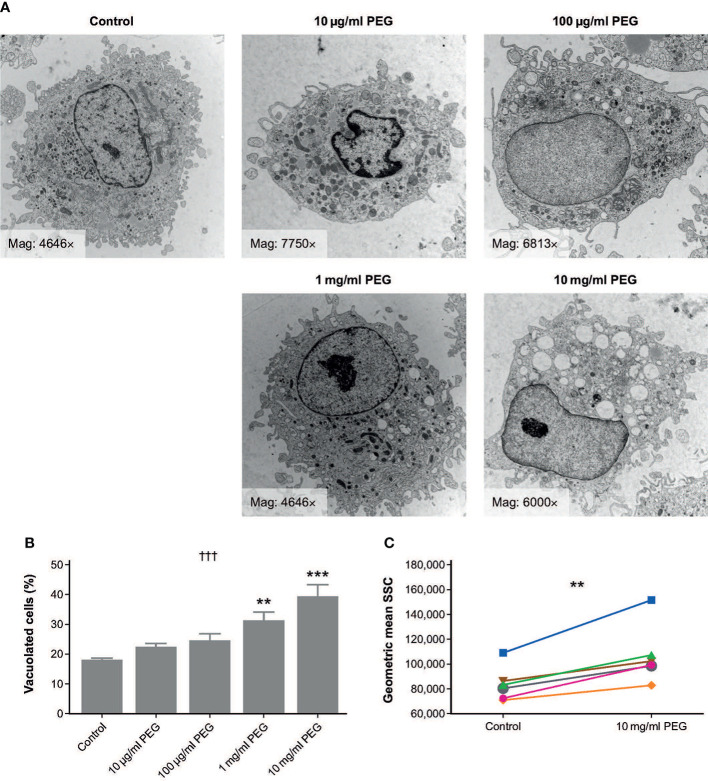
Effect of 24-hour exposure to 20-kDa PEG on vacuolation of human MDMs. **(A)** Representative TEM micrographs of MDMs from a single donor. **(B)** Mean (SD) frequency of vacuolated cells as assessed by light microscopy (n = 3). †††*p* = 0.0002 (one-way ANOVA/Friedman’s test); **p < 0.01, ***p < 0.001 versus control (*post-hoc* test: Dunnett’s multiple comparison). **(C)** Granularity of MDMs assessed by flow cytometry (n = 6). **p = 0.03 (Wilcoxon test). ANOVA, analysis of variance; MDM, monocyte-derived macrophage; PEG, polyethylene glycol; TEM, transmission electron microscopy; SD, standard deviation; SSC, side scatter channel.

The morphology of MDMs from three random donors that were exposed to 20-kDa PEG for 24 hours was assessed using TEM (**Figure 1A**). Vacuoles were observed in both control and PEG-treated cells; however, the number and size of vacuoles appeared greater in the MDMs exposed to higher PEG concentrations. To quantify this effect, semi-thin sections from the same samples were evaluated using light microscopy, which confirmed a concentration-dependent increase in frequency of vacuolation with PEG exposure (*p* = 0.0002; **Figure 1B**). Significant vacuolation versus controls (17.6%) was seen in the MDMs treated with PEG at the higher concentrations of 1 mg/ml (30.6%; *p* < 0.01) and 10 mg/ml (39.0%; *p* < 0.001). No statistically significant PEG-mediated vacuolation was detected in the MDMs exposed to PEG concentrations of up to 100 μg/ml.

MDMs from six random donors were also assessed for changes in morphology after 24-hour exposure to 10 mg/ml PEG using flow cytometry. Geometric mean SSC was significantly higher in PEG-treated cells than in controls, indicating increased granularity (*p* = 0.0033; **Figure 1C**).

### PEG uptake by MDMs

**Figure 2:**
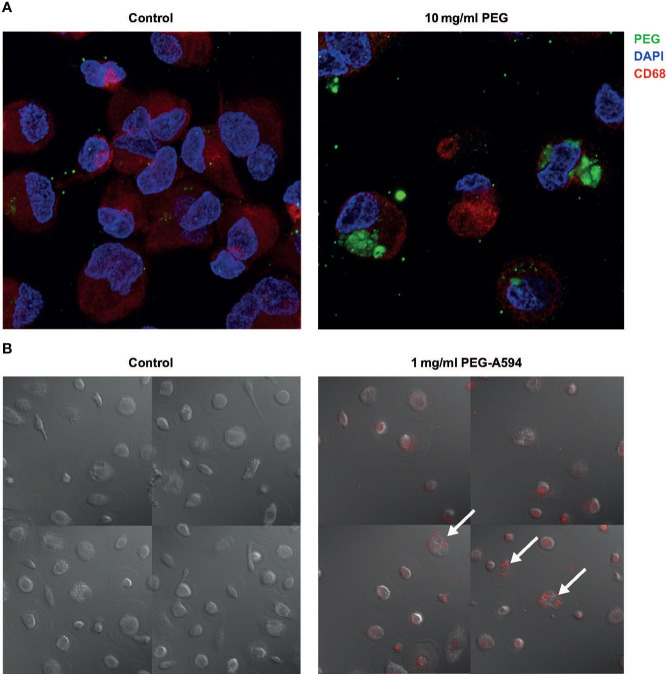
Uptake of 20-kDa PEG in human MDMs. **(A)** Representative confocal images of control MDMs and those exposed to PEG for 24 hours (n = 3). **(B)** Final images from live-cell imaging of MDMs from a single donor (30 hours after addition of PEG-A594). Arrows highlight representative MDMs with localization of PEG-A594 (red) in cytoplasm. DAPI, 4’,6-diamidino-2-phenylindole; MDM, monocyte-derived macrophage; PEG, polyethylene glycol.

To determine whether MDMs actively take up PEG molecules, MDMs from three random donors were exposed to 20-kDa PEG for 24 hours and evaluated using IF. PEG molecules were detected in the cytoplasm of MDMs (CD68+) exposed to 10 mg/ml PEG (**Figure 2A**). The intracellular PEG appeared to be accumulated in clusters or aggregates. To further visualize PEG uptake, live-cell imaging of MDMs from one donor treated with 1 mg/ml PEG-A594 was performed over 26 hours, which confirmed intracellular localization of PEG in MDMs (**Figure 2B**).

### Effect of PEG exposure on phagocytic activity of MDMs

**Figure 3:**
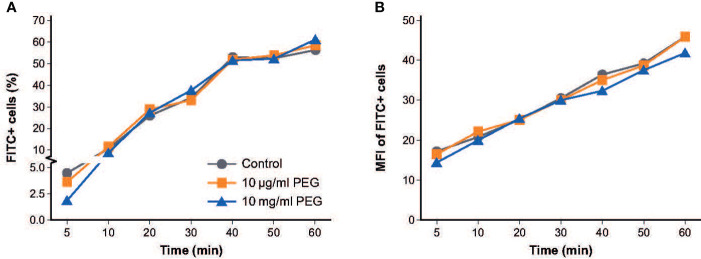
Effect of 20-kDa PEG exposure on phagocytic activity of human MDMs. **(A)** Percentage of MDMs that ingested FITC+ *E. co*li after 24 hours of PEG exposure (n = 3). **(B)** Mean fluorescent intensity of FITC+ MDMs (n = 3). FITC, fluorescein isothiocyanate; MDM, monocyte-derived macrophage; MFI, mean fluorescent intensity; PEG, polyethylene glycol.

To determine the effect of PEG exposure on the phagocytic activity of MDMs, MDMs from three random donors were exposed to 20-kDa PEG for 24 hours at concentrations of 10 μg/ml or 10 mg/ml before the ability of the MDMs to ingest fluorescent bacteria (fluorescein isothiocyanate [FITC]-labeled *E. coli*) over 1 hour was examined. Overall, no relevant effect on the percentage of MDMs that ingested FITC+ *E. coli* was observed at either PEG concentration versus controls (**Figure 3A**). The decrease in phagocytosis activity after short bacteria incubation (5-10 min) for the 10 mg/ml PEG treated MDM group was significant (p-value: <0.0278), whereas no significant decrease of phagocytic activity could be observed for the 10 μg/ml PEG treated MDM group. Mean fluorescent intensity of FITC+ MDMs was similar across the three treatment groups, indicating similar amounts of fluorescent bacteria being engulfed (**Figure 3B**).

### Effect of PEG exposure on the cytokine profile of MDMs

**Figure 4:**
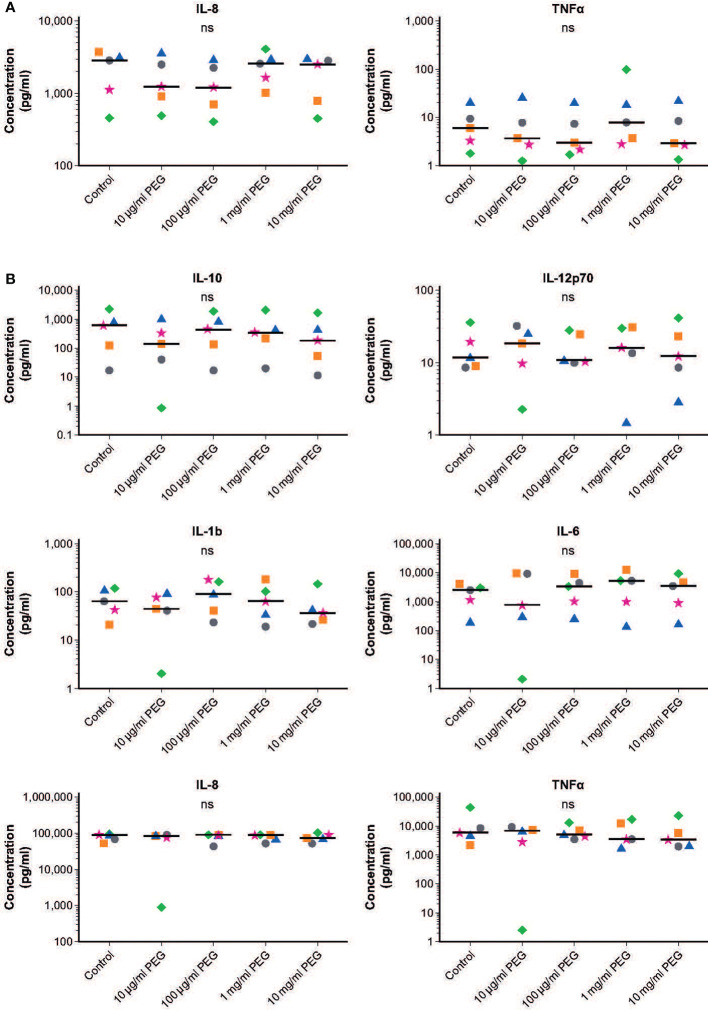
Effect of 20-kDa PEG exposure on cytokine secretion by human MDMs. **(A)** IL-8 and TNFα produced by resting MDMs after 24 hours of PEG exposure (n = 5). Data for not plotted owing to very low expression levels. **(B)** Cytokines produced by LPS-stimulated MDMs after 24 hours of PEG exposure (n = 5). Data shown as individual values, with the horizontal line indicating the median. ^ns^not significant (one-way ANOVA/Friedman’s test; *post-hoc* test: Dunn’s multiple comparison). ANOVA, analysis of variance; IL, interleukin; LPS, lipopolysaccharide; MDM, monocyte-derived macrophage; PEG, polyethylene glycol; TNFα, tumor necrosis factor alpha.

Resting MDMs expressed high levels of IL-8 and low levels of TNFα which did not change upon exposure to PEG (**Figure 4A**). Baseline levels of IL-10, IL-12p70, IL-1β and IL-6 in resting MDMs were very low in absence and presence of PEG in all concentrations tested (**Supplementary Figure 2**). Quantitative comparison to baseline cytokine levels in resting MDMs in absence and presence of PEG showed an increased production of these cytokines following treatment with LPS (data not shown). No significant effects of 24-hour exposure to 20-kDa PEG on cytokine secretion were found in resting or LPS-stimulated MDMs from five random donors.

Similar responsiveness to LPS stimulation was observed in control and PEG-treated MDMs, with increased production of IL-10, IL-12p70, IL-1β, IL-6, IL-8 and TNFα (**Figures 4B** and **Supplementary Figure 2**). Almost no cytokines were detected for one donor in the MDMs treated with 10 μg/ml PEG; this was considered a sample artifact.

### Effect of PEG exposure on expression of MDM surface markers

**Figure 5:**
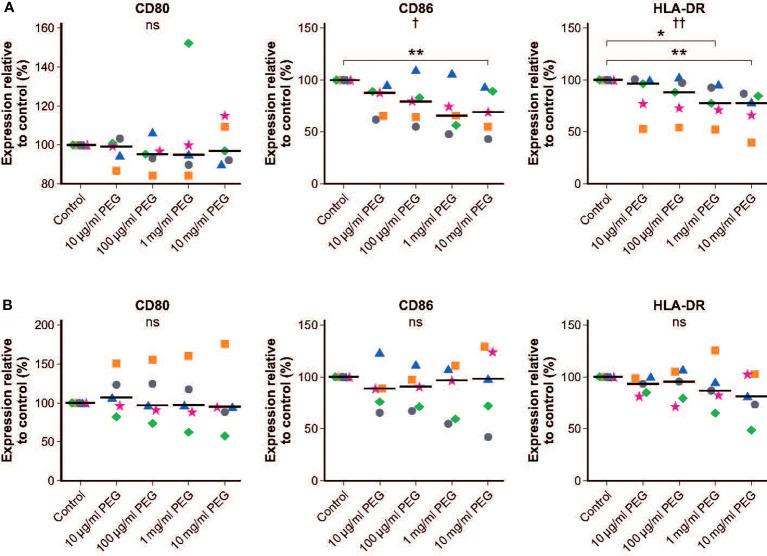
Effect of 20-kDa PEG exposure on cell surface markers of human MDMs. **(A)** Expression in resting MDMs after 24 hours of PEG exposure (n = 5). **(B)** Expression in LPS-stimulated MDMs after 24 hours of PEG exposure (n = 5). Data shown as individual values relative to control, with the horizontal line indicating the median. †*p* = 0.0229, ††*p* = 0.0024, ^ns^not significant (one-way ANOVA/Friedman’s Test); CD86: **p = 0.0014; HLA-DR: *p = 0.0051, **p = 0.0007 versus control (*post-hoc* test: Dunn’s multiple comparisons). ANOVA, analysis of variance; LPS, lipopolysaccharide; MDM, monocyte-derived macrophage; PEG, polyethylene glycol.

No significant effect of 24-hour exposure to 20-kDa PEG on expression of CD80 was seen in resting MDMs from five random donors. There was a slight but significant concentration-dependent decrease in the expression of CD86 (*p* = 0.0229) and HLA-DR (*p* = 0.0024) in resting MDMs exposed to PEG (**Figure 5A**). This was significant versus controls only in the MDMs incubated with PEG at concentrations of at least 10 mg/ml (CD86) or at least 1 mg/ml (HLA-DR). **Table 1A** shows mean fluorescent intensities (MFI) of surface activation markers CD80, CD86 and HLA-DR of unstimulated MDMs in absence and presence of increasing concentrations of PEG.

**Table 1:** Effect of PEG exposure on expression of human MDM surface markers.

(A)					
	mean MFI				
	no PEG	10µg/ml PEG	100µg/ml PEG	1mg/ml PEG	10mg/ml PEG
CD86	9982	6323	5741	5044	4578
CD80	1104	1048	1006	1068	1115
HLA-DR	6424	6052	5844	5540	5169
(B)					
	**x-fold change from resting to LPS-activated MDMs**				
	**no PEG**	**10µg/ml PEG**	**100µg/ml PEG**	**1mg/ml PEG**	**10mg/ml PEG**
CD86	1,30	1,39	1,59	1,54	1,44
CD80	4,21	5,40	5,60	5,15	4,74
HLA-DR	1,25	1,23	1,29	1,28	1,17

**(A)** Mean fluorescent intensity (MFI) of unstimulated MDMs in absence and presence of increasing concentrations of PEG. **(B)** Response rates upon stimulation of MDMs by LPS in absence and presence of increasing concentrations of PEG.

Similar responsiveness to LPS stimulation was observed in control and PEG-treated MDMs, with increases in the mean expression levels of CD80, CD86 and HLA-DR in controls of 4.21-fold, 1.30-fold and 1.25-fold, respectively. No significant effects of PEG exposure on expression of surface markers in LPS-activated MDMs was observed (**Figure 5B**). **Table 1B** shows response rates upon stimulation in absence and presence of increasing concentrations of PEG relative to resting cells to determine how the expression patterns change in the presence of LPS and to show the magnitude of the effect.

### Effect of PEG exposure on MDM viability

**Figure 6:**
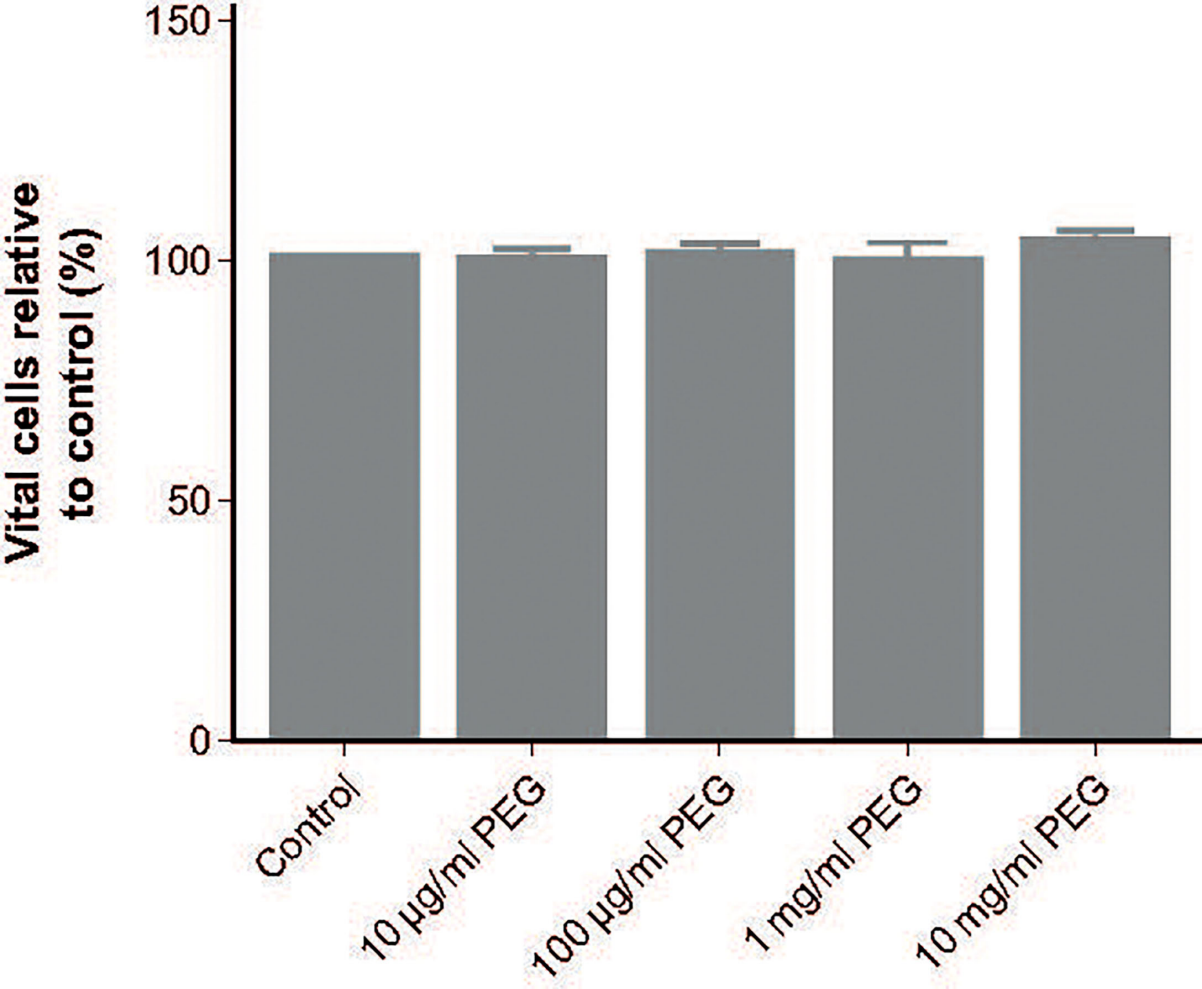
Effect of 20-kDa PEG exposure on viability of human MDMs. Mean (SD) vital cells (negative for Annexin V and propidium iodide) after 24 hours of PEG exposure, relative to control (n = 5). MDM, monocyte-derived macrophage; PEG, polyethylene glycol; SD, standard deviation.

Cell viability following 24-hour exposure to 20-kDa PEG was assessed in MDMs from five random donors. No impact of PEG exposure on viability was observed, with all groups having a similar percentage of vital cells to controls (76.0%; **Figure 6**).

## Discussion

PEGylation of drug molecules, such as clotting factor concentrates, results in extended half-life products that require less frequent dosing than standard half-life drugs (2). These products therefore have the potential to reduce treatment burden and improve adherence in people on chronic prophylactic therapy, as is the standard of care in hemophilia (4, 5). The potential for PEG to cause vacuolation of phagocytic cells requires investigation to inform understanding of the safety of PEGylated therapies. This study evaluated the effects of *in vitro* exposure of human MDMs to the 20-kDa PEG contained within rurioctocog alfa pegol (rFVIII-PEG) at concentrations ranging from 10 μg/ml to 10 mg/ml. Results indicate that PEG-mediated vacuolation is concentration-dependent, with significant vacuolation only occurring with exposure to extremely high PEG concentrations, far above levels reached even upon high-dose therapy with PEGylated drug substances. Functional assays demonstrated that, even with those high PEG concentrations, vacuolated MDMs maintain viability and functionality after PEG exposure.

Vacuolation of macrophages is part of the normal cellular process for removing foreign materials and is a common finding in animal cells (6). Correspondingly, vacuoles were observed in 17.6% of control MDMs, which were not exposed to PEG. Significant vacuolation of MDMs versus controls only occurred after 24-hour exposure to concentrations of PEG of at least 1 mg/ml. This is approximately 10,000-fold higher than the calculated circulating concentration of PEG in patients after receiving a single prophylactic dose of rurioctocog alfa pegol 80 IU/kg (~0.1 μg/ml) (2). No significant vacuolation was observed with PEG concentrations of up to 100 μg/ml. As anticipated, PEG molecules were observed to be taken up by the MDMs. The presence of aggregates of PEG within the cytoplasm indicates that the ingested PEG molecules were potentially located within vacuoles; however, this possibility could not be clarified in this study. It has been hypothesized that PEG taken up by phagocytic cells is released into the circulation by exocytosis or during apoptosis, and is then eliminated *via* the kidneys or liver (2). The present study was limited to investigation of the effect of PEG on human MDMs. It remains subject to further investigation if the results could be extrapolated to other types of phagocytic cells like dendritic cells or Kupffer cells, although it is believed that all these cells share similar mechanisms of endocytosis.

The primary function of macrophages is to engulf and destroy pathogens and dying cells *via* phagocytosis (14). No impairment of MDM phagocytic activity was observed with 24-hour exposure to 20-kDa PEG, even at concentrations associated with significant vacuolation. This is in line with macrophage vacuolation representing an adaptive response to an increased demand for clearance of PEG, rather than a toxic effect (7). In support of this conclusion, viability of MDMs was not affected by exposure to PEG.

As well as playing a key role in the innate immune system, phagocytic macrophages also initiate and direct the adaptive immune response by triggering T-cell activation and differentiation through antigen presentation and the release of specific cytokines (14, 15). Analyses of key cell surface markers for antigen presentation did not identify any significant effects of PEG exposure on expression levels in LPS-stimulated MDMs. In resting MDMs exposed to PEG, there was a slight but significant concentration-dependent decrease in the expression of HLA-DR (MHC class II) and the co-stimulatory molecule CD86 versus controls. This was only significant at very high PEG concentrations and did not appear to have any functional consequences as PEG-treated MDMs exhibited normal responsiveness to LPS stimulation. Furthermore, there was no effect of PEG exposure on the expression of the cytokines IL-1β, IL-6, IL-8, IL-10, IL-12p70 and TNFα in resting or stimulated MDMs at any of the doses tested. Together, these findings support previous studies demonstrating the safety of exposure to PEG polymers of 20 kDa and larger in mammals, even at supra-physiological doses (2, 7). In rats administered a single supra-therapeutic dose of radiolabeled rurioctocog alfa pegol, all radioactivity was completely eliminated over 6 weeks, with urine as the major excretion route (2). In a 3-month, repeated-dose study in rats that received 10-, 20- or 40-kDa PEG intravenously at a dose of 100 mg/kg, no adverse events were observed in any group and there was no vacuolation detected in the animals exposed to 10- or 20-kDa PEG (7). Immunohistochemical staining of PEG was, however, found in vacuoles in macrophages and choroid plexus epithelial cells from the rats that received 40-kDa PEG (7). In a long-term study of a recombinant PEGylated coagulation factor IX conjugated to 40-kDa PEG in immune-deficient rats, PEG was found in vacuoles of choroid plexus epithelial cells in a dose-dependent manner (16). Conversely, the same 40-kDa PEG attached to a recombinant truncated coagulation factor VIII protein did not induce vacuolation of choroid plexus epithelial cells in a similarly designed study (17). This highlights the drug molecule to which PEG is attached as well as the PEG molecule size as key determining factors for vacuolation risk. Several studies with different molecular weight PEGs covering a broad molecular weight range up to 190 kDa have shown that uptake and vacuole formation increased with an increase in the PEG molecular weight (6, 18). We can therefore assume that in our experimental setup significant vacuole formation would similarly occur with PEGs larger than 20 kDa. However, although likely, our study cannot predict if MDMs exposed to larger PEGs would retain their phagocytic activity similar to MDMs with vacuoles induced by 20 kDa PEG. It is a general question if it is possible extrapolating results from *in vivo* animal studies with high systemic PEG exposure or *in vitro* incubation studies as the one presented here to regular long-term PEG exposure in humans. Despite the limitations the European Medicines Agency Committee for Medicinal Products for Human Use concluded that there is a risk of vacuolation of ependymal cells only with PEG sizes of 40 kDa or greater and cumulative exposure of 0.4 μmol/kg/month (18). No safety concerns associated with 20-kDa PEG have been identified at clinically relevant dose levels (2). Furthermore, the most recent World Federation of Hemophilia guidelines state that there is no evidence of clinical safety issues with the use of PEGylated factor products in people with hemophilia (5).

## Conclusion

Vacuolation of human MDMs after exposure to 20-kDa PEG only occurred with PEG concentrations far in excess of those equivalent to clinically relevant doses of rurioctocog alfa pegol. Vacuolated MDMs maintained viability and functionality after PEG exposure, supporting vacuolation as an adaptive rather than toxic effect.

## Data availability statement

The raw data supporting the conclusions of this article will be made available by the authors, without undue reservation.

## Author contributions

AS designed the study, conducted the experiments, analyzed and interpretated the data. KJ: analyzed and interpretated the data. BR designed the study, and reviewed and interpretated the data. FH supervised the study, interpretated the data, and authored the study report. PT reviewed and interpretated the data, and prepared the manuscript. All authors contributed to critical review of this report and approved the final version.

## Acknowledgments

The authors thank Prof. Dr Sebastien Bachmann (Charité - Universitätsmedizin Berlin, Berlin, Germany) for preparation of samples for TEM and performing TEM, Prof. Dr Andreas Hocke (Charité - Universitätsmedizin Berlin, Berlin, Germany) for performing confocal microscopy and Prof. Dr Hans-Dieter Volk (Director BIH Center for Regenerative Therapies and Institute of Medical Immunology, Charité Universitätsmedizin, Berlin, Germany) for enabling the work at his institute, supervising the collaboration, his helpful review when drafting the manuscript, and responding to comments during the review process. Medical writing was provided by Sarah Graham, PhD of PharmaGenesis London, London, UK, with funding from Takeda.

## Conflict of interest

At the time of the study, BR and FH were full-time employees of Baxalta Innovations GmbH. PT is an employee of Baxalta Innovations GmbH, a Takeda company, and holds relevant Takeda patents and Takeda stocks.

The remaining authors declare that the research was conducted in the absence of any commercial or financial relationships that could be construed as a potential conflict of interest.

The authors declare that this study received funding from Baxalta Innovations GmbH. The funder had the following involvement in the study: study monitoring, quality audits of study site and data, interpretation of data, writing of this article and the decision to submit it for publication.

## Publisher’s note

All claims expressed in this article are solely those of the authors and do not necessarily represent those of their affiliated organizations, or those of the publisher, the editors and the reviewers. Any product that may be evaluated in this article, or claim that may be made by its manufacturer, is not guaranteed or endorsed by the publisher.

## Supplementary material

Supplementary Figure 1 Semi-quantitative Analysis of Vacuolation. Semi-thin sections of MDMs exposed to PEG for 24 h were analyzed with light microscopy. This figure shows a representative image from one sample, demonstrating semi-quantitative analysis (magnification:63x). White dot: marks single MDM; colored dots/circles define different vacuolation stages; green dotted = stage **(A)**: 1 to 3 small vacuoles in a single MDM; magenta dashed = stage **(B)**: > 3 small vacuoles or at least one big vacuole (≈ 5% of cell caliber) in a single MDM; blue solid = stage **(C)**: vacuolation of ≥75% of cell volume. The table below summarizes the statistics for this picture.

Supplementary Figure 2 Effect of 20-kDa PEG exposure on basal cytokine secretion by human MDMs. Cytokines IL-10, IL-12p70, IL-1β and IL-6 produced by resting MDMs after 24 hours of PEG exposure (n = 5). Data shown as individual values, with the horizontal line indicating the median. ns, not significant (one-way ANOVA/Friedman’s test; post-hoc test: Dunn’s multiple comparison). Some values from donors were under detection limit and could therefore not be plotted. ANOVA, analysis of variance; IL, interleukin; LPS, lipopolysaccharide; MDM, monocyte-derived macrophage; PEG, polyethylene glycol.
