# Supplementary material for: Polyethylene glycol 20 kDa-induced vacuolation does not impair phagocytic function of human monocyte-derived macrophages
Source: Front Immunol. 2022 Jul 28;13:894411. doi: 10.3389/fimmu.2022.894411 (PMC9366735; doi:10.3389/fimmu.2022.894411)
Supplement: Supplementary Figure 1 — Semi-quantitative Analysis of Vacuolation. Semi-thin sections of MDMs exposed to PEG for 24 h were analyzed with light microscopy. This figure shows a representative image from one sample, demonstrating semi-quantitative analysis (magnification:63x). White dot: marks single MDM; colored dots/circles define different vacuolation stages; green dotted = stage (A): 1 to 3 small vacuoles in a single MDM; magenta dashed = stage (B): > 3 small vacuoles or at least one big vacuole (≈ 5% of cell caliber) in a single MDM; blue solid = stage (C): vacuolation of ≥75% of cell volume. The table below summarizes the statistics for this picture. [file Presentation_1.pdf]

SUPPLEMENTARY FIGURE 1
Semi-quantitative Analysis of Vacuolation.

Semi-thin sections of MDMs exposed to PEG for 24 h were analyzed with light microscopy. This figure shows a representative image from one sample, demonstrating semi-quantitative analysis (magnification:63x).

White dot: marks single MDM;
colored dots/circles define different vacuolation stages;
green dotted = stage (A): 1 to 3 small vacuoles in a single MDM;
magenta dashed = stage (B): > 3 small vacuoles or at least one big vacuole (≈ 5 % of cell caliber) in a single MDM;
blue solid = stage (C): vacuolation of ≥75% of cell volume.
The table below summarizes the statistics for this picture.

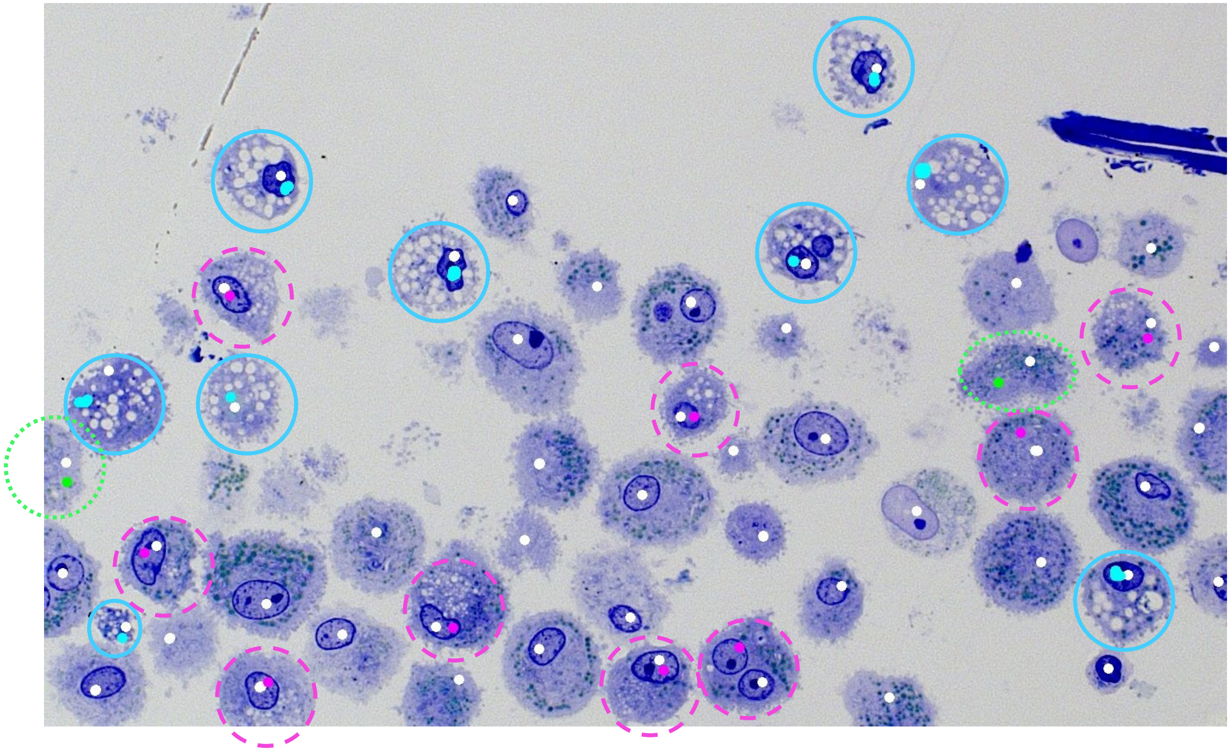

	counts
Total MDMs	51
Stage A	2
Stage B	9
Stage C	9
Vacuolated cells, total	20
Vacuolated cells, %	39.2

SUPPLEMENTARY FIGURE 2

Effect of 20-kDa PEG exposure on basal cytokine secretion by human MDMs.

Cytokines IL-10, IL-12p70, IL-1β and IL-6 produced by resting MDMs after 24 hours of PEG exposure (n = 5). Data shown as individual values, with the horizontal line indicating the median.

^{ns}not significant (one-way ANOVA/Friedman's test; post-hoc test: Dunn's multiple comparison).

Some values from donors were under detection limit and could therefore not be plotted.

ANOVA, analysis of variance; IL, interleukin; LPS, lipopolysaccharide; MDM, monocyte-derived macrophage; PEG, polyethylene glycol.

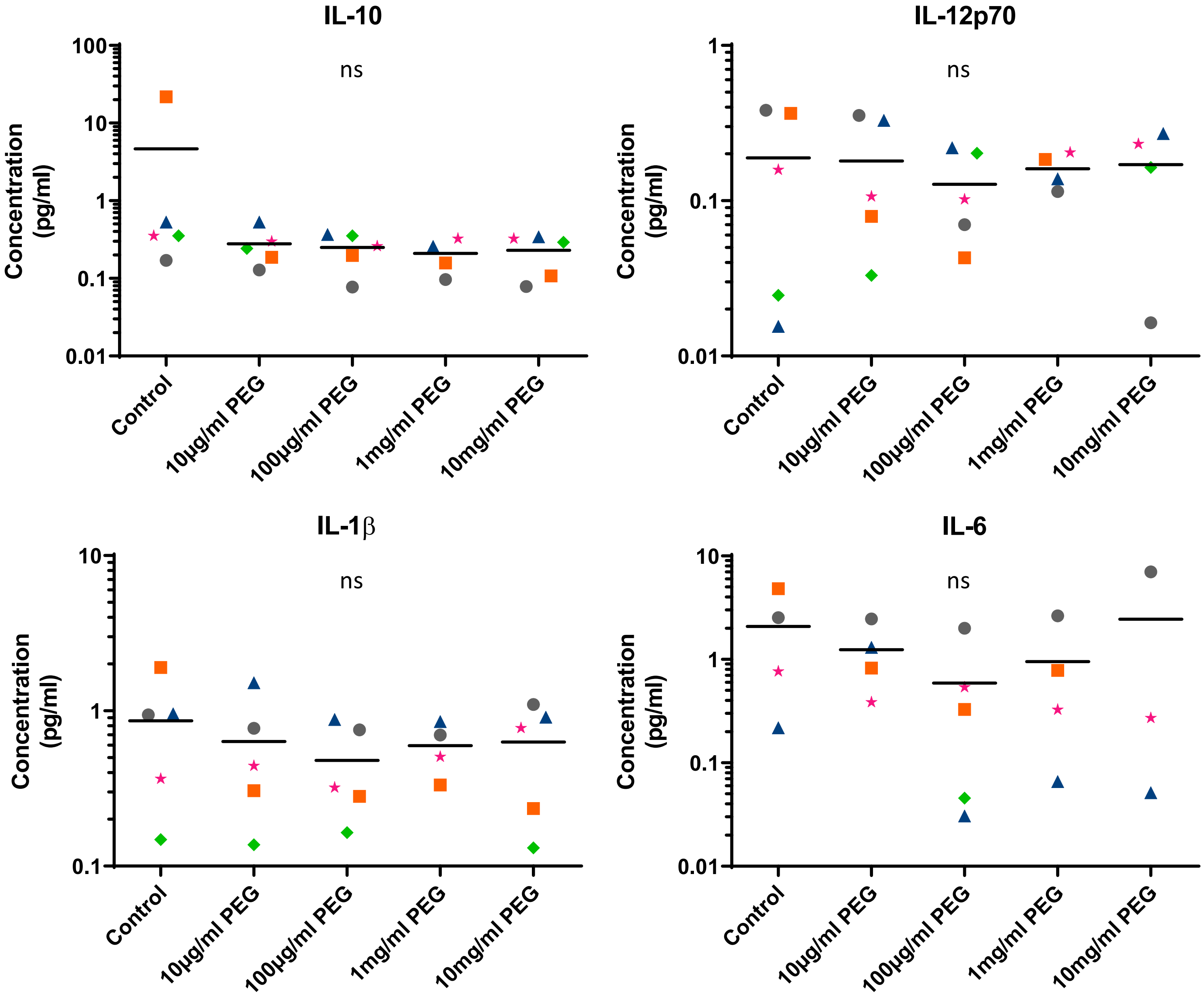

IL-10
ns
IL-12p70
ns
IL-1β
ns
IL-6
ns
Concentration (pg/ml)
0.01
0.1
1
10
100
Control
10µg/ml PEG
100µg/ml PEG
1mg/ml PEG
10mg/ml PEG
